# Macrophage states and immunometabolic adaptation in peritoneal metastases from colorectal cancer

**DOI:** 10.3389/fimmu.2026.1974770

**Published:** 2026-09-28

**Authors:** Weihao Chen, Jiali Wei, Yi Wang, Long Sun, Yang Ye, Kang Sun

**Affiliations:** 1 Department of Colorectal Surgery, The Affiliated Hospital of Jiangsu University, Zhenjiang, China; 2 Department of Infectious Diseases, The Third Hospital of Zhenjiang Affiliated to Jiangsu University, Zhenjiang, China; 3 Medical School of Jiangsu University, Jiangsu University, Zhenjiang, Jiangsu, China

**Keywords:** adoptive cell therapy, colorectal cancer, immune checkpoint blockade, immunometabolism, macrophage states, peritoneal metastasis

## Abstract

Immunotherapy responses in colorectal cancer peritoneal metastases (CRCPM) are not determined by mismatch repair (MMR) status alone. Most MMR-proficient/microsatellite-stable (pMMR/MSS) tumors have low baseline sensitivity, while MMR-deficient/microsatellite instability-high (dMMR/MSI-H) tumors do not respond uniformly, particularly in patients with ascites. This Mini Review compares evidence across compartments and disease stages: human associations, macrophage-resolved functional studies and interventions altering immunotherapy efficacy in mice. Human ascites and tissue studies identify resident-like and monocyte-associated macrophages, including C1Q^+^ and SPP1^+^ states, but shared markers do not establish equivalent functions or origins. During niche conditioning and early seeding, ABCA9-dependent cholesterol handling, tumor-derived lysophosphatidic acid signaling and TLR4–CXCL10 activation can support local T-cell-mediated tumor control, although these studies did not test immune checkpoint blockade (ICB) or adoptive cell therapy (ACT). In established disease, SPP1-associated macrophage-intrinsic peroxisome proliferator-activated receptor-γ (PPARγ) programming and extracellular SPP1–CD44 signaling restrain CD8^+^ T cells, and SPP1 perturbation improves anti-PD-1 and ACT responses in mice. The relative contributions of these routes to treatment rescue remain unresolved. TIM-4 and MerTK provide distinct non-metabolic constraints. Human evidence for the resistance-associated SPP1 state remains cross-sectional. Lipid-related pathways in CRCPM models thus have opposing immune effects rather than acting as a single metabolic switch.

## 1 Introduction

Peritoneal dissemination differs from hematogenous metastasis. Tumor cells occupy the mesothelial surface, adipose-rich omentum, peritoneal fluid and, in advanced disease, malignant ascites. Vascular access, stroma, nutrients and immune composition vary across these connected compartments, exposing tumor-associated macrophages (TAMs) to different local conditions. Peritoneal metastases from colorectal cancer (CRC), hereafter CRCPM, are managed with selected systemic and locoregional treatments despite uneven supporting evidence (1). Peritoneal lesions are often stroma-rich and resemble consensus molecular subtype 4 (CMS4), but no single genotype defines the site (2).

These anatomical differences place macrophages at the intersection of local metabolism and treatment response, but they also complicate interpretation. A cell recovered from ascites may differ in lineage, exposure and function from a marker-matched cell in an omental nodule. Studies published through August 2026 assess different endpoints. Human datasets associate macrophage states with disease. Several macrophage-resolved experiments show altered T-cell function but do not test ICB or ACT, while a smaller set of mouse studies finds that targeting SPP1, TIM-4, MerTK or CSF1R changes treatment efficacy. These are differences in experimental endpoint, not fixed molecular categories. Molecular subgroup, compartment, cellular origin and disease stage must therefore be considered when distinguishing the intrinsic low sensitivity of pMMR/MSS disease from primary non-response or acquired resistance.

## 2 Clinical and compartmental context of immunotherapy responses

Associations between peritoneal involvement and immunotherapy response need to be interpreted within mismatch repair/microsatellite instability (MMR/MSI) strata. First-line pembrolizumab prolonged progression-free survival (PFS) in mismatch repair-deficient/microsatellite instability-high (dMMR/MSI-H) metastatic CRC, while nivolumab plus low-dose ipilimumab produced durable responses in a phase II cohort (3, 4). By contrast, ICB activity is generally limited in pMMR/MSS disease: atezolizumab, with or without MEK inhibition, did not improve survival over regorafenib, and regorafenib plus nivolumab showed only modest activity (5, 6). Because none of these trials isolated CRCPM, they define genotype-related response probabilities rather than a macrophage explanation for poor responsiveness at the peritoneal site. Within this genotype-defined background, metastatic distribution and ascites provide additional, although less certain, information. In an MSS series of regorafenib-based ICB combinations, patients with liver or peritoneal metastases had no objective responses, whereas responses were more frequent among patients with lung-limited disease (7). The retrospective, regimen-specific design leaves several alternatives open, including disease burden and coexisting organ sites. Ascites provides a more consistent signal in dMMR/MSI-H disease. Patients with peritoneal metastases but no ascites had outcomes similar to those without peritoneal disease, whereas the adverse association was largely confined to patients with ascites (8). A smaller MSI-H series reported numerically lower response and shorter PFS with peritoneal metastases, without separately analyzing ascites (9). This qualification matters because patients with isolated peritoneal carcinomatosis had a 46% objective response rate in the BIG-RENAPE cohort, and national-database studies associated immunotherapy with longer survival in MSI-H CRCPM (10–12).

This pattern therefore narrows the question: does the peritoneal environment modify genotype-defined sensitivity, and can macrophages help explain that shift? A humanized MSI-H organoid model reproduced organ-site divergence: combined PD-1/CTLA-4 blockade controlled hepatic metastases and the primary tumor but not peritoneal lesions (13). Resistant deposits lacked B cells and tertiary lymphoid structures and contained dysfunctional T cells, although the model did not assign these features to macrophages. Spatial heterogeneity was also evident in one pembrolizumab-treated patient. Peritoneal and left ovarian lesions regressed, whereas omental and right ovarian lesions resisted and contained CD163-marked macrophages together with SPP1-associated programs (14). This single-patient comparison cannot establish a recurrent or causal resistance mechanism. The available evidence instead indicates that response probability varies with molecular subgroup and ascites status, rather than supporting a universal peritoneal sanctuary.

## 3 Macrophage compartments, origins and states

### 3.1 Compartment, origin and state architecture

“Peritoneal macrophage” often denotes a sampling location rather than a lineage. In steady-state mouse models, large F4/80^hi^ and smaller major histocompatibility complex class II^hi^ macrophages differ in developmental origin and responsiveness (15), while retinoic acid-dependent GATA6 maintains cavity localization and tissue programming (16). Fate mapping shows that embryonic and bone-marrow-derived cells coexist in age-dependent proportions (17); resident cells also use local fuels differently from macrophages cultured in standard media (18). This framework cannot assign a function to a macrophage recovered from malignant ascites or a metastatic nodule. Human studies underscore why sampling site matters. Untreated malignant ascites contains more macrophages than reference primary tumors, and inferred communication networks place these cells near the center of the fluid ecosystem (19). The fluid compartment is not uniform: ascites-derived cells collected before and after chemotherapy vary markedly among patients and treatment contexts (20). Solid lesions likewise contain different myeloid and lymphoid mixtures across tissue cohorts and humanized models (21–23), while smaller multivisceral and paired primary–metastasis datasets identify location-specific programs without resolving lineage (24, 25). Marzano et al. (26) compared matched peritumoral samples from primary colorectal tumors and synchronous colorectal liver metastases (CRLM). CRLM was enriched in large, proliferative CTLA4^+^ immunoregulatory macrophages, whereas inflammatory TAMs, including populations expressing the checkpoint ligands PD-L1 and NECTIN2, were more frequent around the primary tumor. In the same study, a higher frequency of CD163^+^PCNA^+^ macrophages in peritumoral liver tissue surrounding CRLM was associated with shorter disease-free survival. This comparison places peritoneal macrophage heterogeneity within a broader pattern of tissue-specific myeloid remodeling in metastatic CRC.

Spatial profiling links macrophage-rich, fibrocollagenous regions to T-cell exhaustion, and bulk transcriptomic analyses find macrophage and tumor necrosis factor (TNF) signatures enriched in CMS4 disease (27, 28). These analyses describe co-location and inferred abundance, not direction of effect. Fibroblasts may help stabilize local states through tissue-specific myeloid interactions and an IGFBP2–CD38-associated circuit (29, 30). In a small prospective cohort, fluorouracil, leucovorin, oxaliplatin and irinotecan (FOLFOXIRI) plus bevacizumab altered immune composition more clearly in blood and peritoneal fluid than in tissue (31). The pattern illustrates how apparent remodeling may partly reflect the compartment sampled. Time-resolved mouse studies begin to distinguish recruitment from local expansion. Ferriz et al. (32) linked a low-inflammatory peritoneal milieu to limited monocyte recruitment and differentiation. In the same model, resident macrophages co-expressing T-cell immunoglobulin and mucin domain-containing protein 4 (TIM-4) and triggering receptor expressed on myeloid cells 1 (TREM1) proliferated, moved toward the omentum and promoted tumor growth. Expression of *Adora2a*, *Adora2b*, *Arg1*, *Ido2* and *Acod1* suggests adenosine sensing, amino-acid metabolism and itaconate-related programming, but does not establish pathway activity or immunotherapy resistance. Dai et al. (33) reported that SPP1+ cavity macrophages were enriched across separate treatment-resistant patient groups, but cross-sectional sampling cannot distinguish therapy-induced change from selection or differences in disease stage. Other mouse studies associated small peritoneal macrophages and interleukin-10 (IL-10)-linked macrophages with progression (34, 35). The IL-10 study used CMT93 tumors in non-obese diabetic/severe combined immunodeficient (NOD/SCID) mice, limiting inference about adaptive immunity. Available cellular maps come from cohorts with different molecular subtypes and treatment exposures (19, 20, 22, 33). Current patient-level data do not yet connect a defined macrophage state with either the ascites-specific adverse outcomes reported in dMMR/MSI-H disease (8) or the numerically lower response and shorter PFS reported in MSI-H peritoneal metastases more broadly (9). Across studies, macrophage nomenclature reflects three different biological dimensions. C1Q^+^ and SPP1^+^ denote transcriptional states; TIM-4, TREM1 and VSIG4 describe surface phenotypes; resident-like and monocyte-associated terms indicate inferred origin. These dimensions may overlap, but their correspondence has not been tested directly in matched CRCPM compartments (**Table 1**).

**Table 1:** Cross-study comparison of macrophage labels used in CRCPM.

Reported label and compartment	Defining features	Inferred origin	Functional evidence
C1Q^+^ CM—human PF/PM	C1QA; MARCO/CD163^bright^/VSIG4; VCAN^-^	Resident-like; not lineage-traced	No state-selective test
C1Q^+^ mono-CM—human PF/PM	C1QA; VCAN/CD163^dim^/VSIG4	Monocyte-associated; not lineage-traced	No state-selective test
SPP1^+^ CM—human PF/PM and murine CRCPM	SPP1; MARCO/CD163^bright^/VSIG4, LYVE1/CD163	Resident-like signatures; lineage unresolved	*Spp1* targeting improved anti-PD-1 and OT-I ACT efficacy
TIM-4^+^TREM1^+^ macrophages—murine cavity/omentum	TIM-4, TREM1	Resident	Tumor growth; ICB/ACT not tested
TIM-4^+^ cavity macrophages—murine MC38/CT26 models	TIM-4, F4/80^hi^, MHC-II^lo^	Resident	TIM-4 perturbation improved anti-PD-1 and OT-I ACT efficacy
VSIG4^+^ macrophages—human PF/PM and murine cavity/omentum	Shared by C1Q^+^ and SPP1^+^ states; most murine VSIG4^+^ LPMs are TIM-4^+^	Resident-enriched, but not lineage-exclusive	VSIG4^+^-cell depletion accelerated early tumor growth; receptor knockout left lavage tumor-cell fractions largely unchanged, with lower tumor-cell fractions in day-16 omental nodules (small cohorts).
Monocyte-associated schemes—human PF/PM or murine cavity	mono-CM, monocyte-derived macrophage and SPM; study-specific definitions	Monocyte-associated	No common state-selective test

PF, peritoneal fluid; PM, peritoneal metastasis; CM, cavity macrophage; mono-CM, monocyte-associated cavity macrophage; MHC-II, major histocompatibility complex class II; LPM, large peritoneal macrophage; SPM, small peritoneal macrophage. These labels are neither mutually exclusive nor directly interchangeable, and no study has aligned all populations across datasets. The SPP1 and TIM-4 experiments tested the corresponding molecules rather than selectively eliminating an entire transcriptional state. The TIM-4 treatment experiments in reference 51 did not specifically test the TIM-4^+^TREM1^+^ state defined in reference 32.

### 3.2 VSIG4 as a separating marker, population and receptor

VSIG4 has been used as a marker, population label and receptor target. Saris et al. used V-set and immunoglobulin domain-containing 4 (VSIG4) to identify C1Q^+^ and SPP1^+^ states in peritoneal fluid and metastases. Mouse tracing in the same study suggested that some lesion macrophages originated in the peritoneal cavity (22). In that setting, VSIG4 helps identify related populations; it does not show that receptor signaling determines their function. Perturbation studies ask different questions. Depleting large VSIG4-expressing cavity-resident macrophages either seven days before MC38 inoculation or two days after inoculation accelerated tumor growth, supporting a protective role during implantation and early growth, not established disease (36). *Vsig4* deletion left the tumor-cell fraction in peritoneal lavages largely unchanged; omental nodules showed a lower tumor-cell fraction at day 16 in small cohorts (37). Human peritoneal-metastasis experiments have separately linked VSIG4 expression or secretion to suppressive paracrine signaling (38). The discrepancy becomes less surprising when cell depletion, receptor deletion, compartment and disease stage are considered together. Receptor-specific blockade should be compared with timed cell depletion after nodules are established. Until then, VSIG4 remains a context-dependent marker rather than a validated therapeutic target. Depleting VSIG4^+^ macrophages has broader effects than deleting the receptor, and a function observed during seeding may not persist in stromal nodules.

## 4 Immunometabolic adaptation

### 4.1 SPP1-associated programs and preclinical treatment resistance

Among direct CRCPM studies, the SPP1 work most clearly connects a human resistance-associated state with intervention experiments that measured treatment efficacy. Single-cell RNA sequencing (scRNA-seq) of ascites from 20 patients showed enrichment of SPP1^+^ cavity macrophages in an ICB/ACT-resistant group (33). A separate cohort of 36 samples analyzed by proteomics and spatial profiling supported the same association. The resistant group was clinically heterogeneous: six resistant cases had received anti-PD-1 therapy, one patient had received chimeric antigen receptor (CAR)-T cells, and one had received CAR-T cells plus CAR-engineered natural killer cells. Because patients were sampled at different treatment stages, the enrichment cannot establish that therapy induced the state. Mouse experiments then provided two mechanistic routes. Within macrophages, SPP1 was associated with a transcriptional program biased toward peroxisome proliferator-activated receptor-γ (PPARγ). *Spp1* loss reduced precursor availability for PPARγ ligand synthesis, increased nuclear factor-κB (NF-κB) activity and restored CD8^+^ T-cell function (33). Rescue with 15-deoxy-Δ12,14-prostaglandin J2 plus fatty acids placed lipid-derived ligands downstream of SPP1. In a parallel experiment, extracellular SPP1 acted through CD44 to suppress CD8^+^ effector function. Myeloid *Spp1* deletion or SPP1 neutralization improved anti-PD-1 therapy in MC38 and CT26 peritoneal models; SPP1 neutralization also enhanced ovalbumin-specific OT-I ACT against ovalbumin-expressing MC38 tumors. These findings position SPP1 as more than a cluster marker, while leaving a key question unresolved. Genetic and antibody interventions altered both macrophage-intrinsic lipid programming and extracellular SPP1–CD44 signaling, so the contribution of each route to therapeutic rescue remains unclear. The study supports a causal role for SPP1 in preclinical ICB and ACT, but acquired resistance in patients still requires longitudinal evidence.

### 4.2 Immune-supportive lipid and cholesterol pathways during niche conditioning and early seeding

These lipid-related pathways were studied before or during early peritoneal seeding rather than in established disease, and none was tested with ICB or ACT. They therefore inform CRCPM biology without, by themselves, demonstrating immunotherapy resistance. Their effects were not uniformly suppressive. In premetastatic adipose tissue, 5-aza-2′-deoxycytidine increased ABCA9-dependent cholesterol accumulation in macrophages, activated p65/interleukin-6 (IL-6) signaling and improved T-cell-dependent tumor control (39). Macrophage or T-cell depletion, as well as genetic or pharmacological perturbation, weakened the effect, supporting a functional macrophage–T-cell interaction in this model. Human evidence came mainly from ex vivo CRC and omental tissue rather than clinical CRCPM outcomes. A second route originates in tumor cells. Reduced *Agpat4* expression increased lysophosphatidic acid (LPA), which engaged LPA receptors 1 and 3 on macrophages and activated p38/p65 signaling. Inflammatory cytokine release and CD4^+^ and CD8^+^ T-cell activity increased, restraining peritoneal implantation (40). The macrophage therefore responded to a tumor-derived lipid signal, but the study did not demonstrate autonomous metabolic reprogramming within the macrophage. Short-term dietary fat exposure produced a similar T-cell-supportive effect during early seeding by increasing Toll-like receptor 4 (TLR4)-dependent phagocytosis and CXCL10-mediated T-cell recruitment by adipose-tissue macrophages (41). Macrophage depletion or pathway blockade reduced protection. The acute timing, however, limits interpretation because these inflammatory states may not persist in established nodules, malignant ascites or repeatedly treated disease.

### 4.3 Interpreting divergent metabolic programs

Studies with incomplete mechanistic endpoints help define how far the metabolic interpretation can go. Macrophage-specific *Mgll* deficiency increased suppressive myeloid features, reduced T-cell representation and accelerated murine CRCPM (42). Transcriptomic analysis implicated TLR, hypoxia-inducible factor 1 (HIF-1) and PD-1/PD-L1 pathways, but CRCPM-specific metabolites, rescue experiments and ICB/ACT outcomes remain untested. Earlier work connected macrophage monoglyceride lipase (MGLL) to cannabinoid receptor 2 signaling across several tumor models (43), providing mechanistic context without CRCPM-specific ICB evidence. Enzyme expression is not synonymous with a metabolic mechanism. Macrophage ABHD5 restrained NF-κB-dependent matrix metalloproteinase expression and metastasis through effects separable from its canonical role in triglyceride metabolism (44). Hydroxygenkwanin altered NF-κB/STAT5 and STAT6/JMJD3/PPARγ-associated macrophage states in MC38 peritoneal disease (45). Without flux measurements, metabolite rescue or ICB testing, however, this finding is better described as state reprogramming. The surrounding niche also changes substrate availability. Fibroblasts, adipose-derived cells and tumor cells alter lipid supply, fatty-acid catabolism, glycolytic support and sialylation (46–49). Resiquimod-loaded nanoparticles produce coordinated metabolic and immune shifts without measuring macrophage-intrinsic flux (50). These studies differ in substrate, cellular source, stage and endpoint; they do not establish a single switch. A stronger causal case requires macrophage-specific perturbation, metabolic readouts, functional rescue and a therapeutic endpoint in the same CRCPM model. No CRCPM study of lactate or lactylation, arginine, adenosine, glycolysis or fatty-acid oxidation has met all four criteria, including an ICB or ACT endpoint.

## 5 Macrophage-associated constraints on ICB and ACT

Only a small subset of studies directly measured ICB or ACT efficacy; most reported macrophage states, tumor growth, or T-cell function without testing either therapy. Myeloid *Spp1* deletion or SPP1 neutralization improved anti-PD-1 efficacy, and neutralization also enhanced OT-I ACT (33). These treatment-level effects cannot yet be attributed to the metabolic route alone. In the CT26 peritoneal metastasis model, CSF1R blockade combined with anti-PD-1 reduced disease burden and extended survival (22). This supports a net contribution from CSF1R-sensitive myeloid cells, including macrophages, but neither identifies a corresponding human state nor excludes concurrent effects on protective macrophages. **Figure 1** summarizes these distinctions; **Supplementary Figure 1** provides the broader compartmental schematic. Similar treatment endpoints can arise through non-metabolic mechanisms. TIM-4^+^ cavity-resident macrophages bound phosphatidylserine on activated CD8^+^ T cells, retained them locally, and limited their proliferation. TIM-4 blockade improved anti-PD-1 therapy in MC38 and CT26 models and OT-I ACT in MC38-OVA tumors (51). Macrophage-directed siMerTK impaired efferocytosis and enhanced PD-1 blockade in colorectal peritoneal and hepatic metastasis models (52), whereas pharmacological MerTK inhibition strengthened a radiation-based immunogenic vaccine (53). Although both pathways involve the handling of activated or dying cells, neither has been placed within the SPP1–PPARγ program.

**Figure 1:**
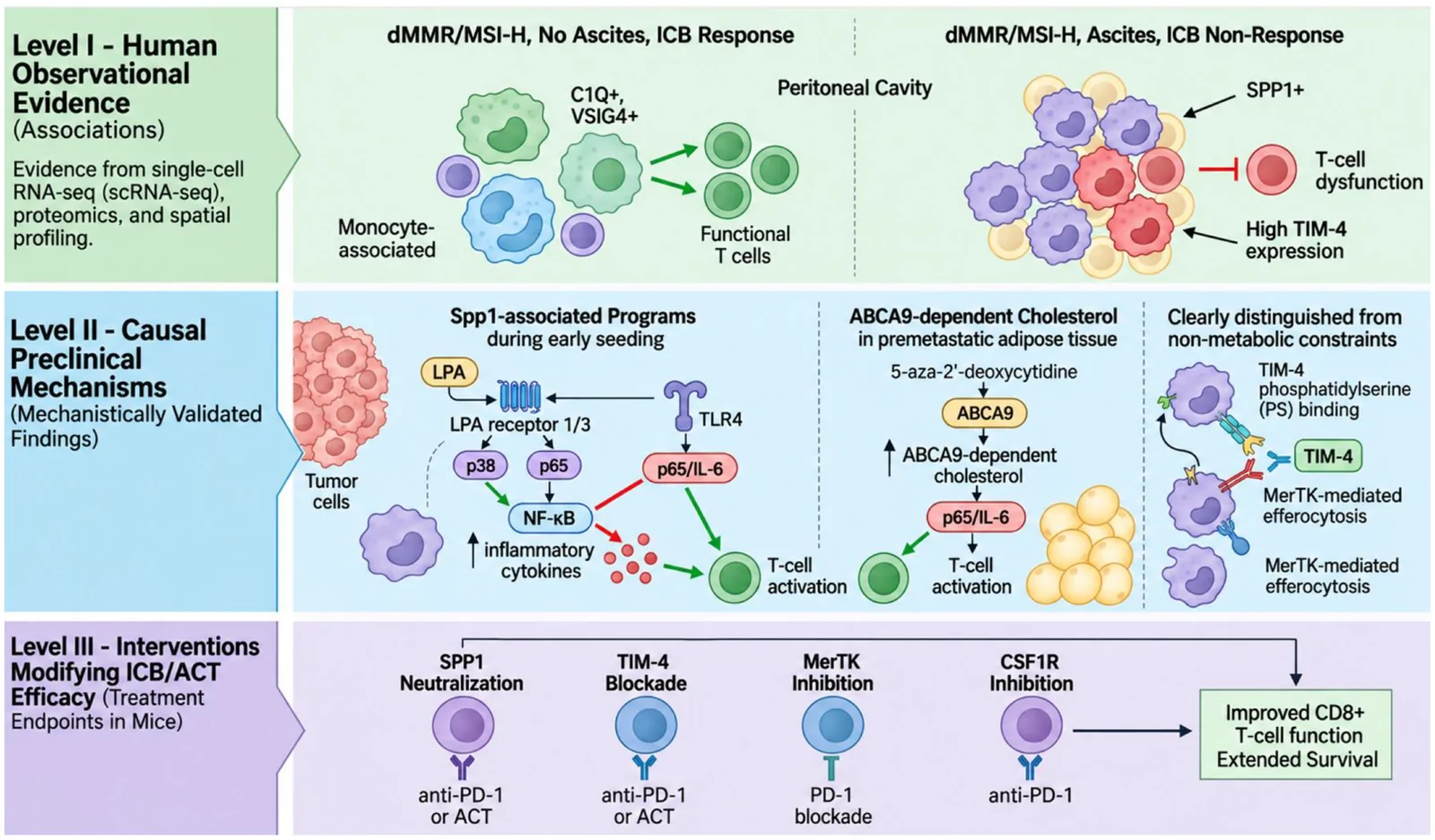
Evidence-stratified links between macrophage states, immunometabolic pathways and treatment response in CRCPM. The figure organizes the evidence into three horizontal levels: (I) human observational associations; (II) preclinical findings that inform mechanism but do not test ICB or ACT; and (III) preclinical interventions that altered ICB or ACT efficacy in mice. At Level I, the dMMR/MSI-H examples with and without ascites place clinical outcome patterns alongside macrophage states from separate cohorts. They therefore do not establish that C1Q+/VSIG4+ or SPP1+/TIM-4-high macrophages were measured in the same patients or that either state caused the clinical outcome. Level II covers early-seeding and niche-conditioning pathways, including tumor-derived LPA–LPAR1/3–p38/p65 signaling, TLR4-associated inflammatory and phagocytic responses, and ABCA9-dependent cholesterol handling. These pathways support T-cell activity in CRCPM models, but none has been tested with ICB or ACT. Level III includes myeloid *Spp1* deletion or SPP1 neutralization with anti-PD-1 or OT-I ACT, TIM-4 blockade with anti-PD-1 or OT-I ACT, MerTK inhibition with PD-1 blockade, and CSF1R inhibition with anti-PD-1. Pharmacological MerTK inhibition in a radiation-based immunogenic-vaccine study is considered separately because that study did not test ICB or ACT. The classification follows the endpoint supported by each evidence link, rather than assigning a fixed category to a molecule or marker-defined population. The layout does not imply a fixed lineage or temporal progression. ACT, adoptive cell therapy; ABCA9, ATP-binding cassette subfamily A member 9; CRCPM, colorectal cancer peritoneal metastases; dMMR/MSI-H, mismatch repair-deficient/microsatellite instability-high; ICB, immune checkpoint blockade; LPA, lysophosphatidic acid; LPAR1/3, lysophosphatidic acid receptors 1 and 3; MerTK, MER proto-oncogene tyrosine kinase; OT-I, ovalbumin-specific CD8+ T cells; SPP1, secreted phosphoprotein 1; TIM-4, T-cell immunoglobulin and mucin domain-containing protein 4; TLR4, Toll-like receptor 4; VSIG4, V-set and immunoglobulin domain-containing 4.

The surrounding niche can also be manipulated without identifying a single macrophage state. Intraperitoneal Toll-like receptor 9 (TLR9) agonism altered the fibroblastic reticular cell–retinoic acid–GATA6 circuit and improved anti-PD-1 efficacy (54). Separate studies improved checkpoint-based tumor control using approaches that included adenoviral PD-L1 blockade with macrophage depletion, oncolytic vaccinia virus, stimulator of interferon genes (STING) activation or heated intraperitoneal chemotherapy (55–58). These combinations show that local ICB efficacy is modifiable; they do not imply a shared macrophage mechanism. Evidence linking macrophages to ACT resistance remains preclinical. Chow and Dai both used ovalbumin-expressing MC38 tumors and antigen-defined OT-I T cells (33, 51); no CRCPM cohort has linked cavity macrophages to ACT outcomes. The inclusion of ACT-treated patients in Dai’s cross-sectional resistant cohort does not resolve this gap. **Table 1** compares reported labels rather than treatment evidence: perturbing SPP1 or TIM-4 altered treatment response in mice but did not establish equivalence among the corresponding marker-positive populations.

## 6 Therapeutic implications and translational priorities

Broad depletion can estimate the aggregate contribution of depletion-sensitive myeloid populations, but its breadth limits therapeutic specificity. CSF1R inhibition and clodronate do not cleanly discriminate among cells with different origins or stage-specific roles. Cavity-retaining nanoparticles improved local clodronate exposure and enhanced oxaliplatin in preclinical peritoneal metastasis (59), yet the intervention still depleted macrophages at the population level. VSIG4^+^ cell depletion accelerated early tumor growth (36), supporting investigation of state-selective treatment, although no CRCPM study has shown that it outperforms depletion. Targets tied to defined macrophage functions may offer greater selectivity, but each poses a different development problem. SPP1-directed strategies should distinguish macrophage-intrinsic lipid programming from extracellular signaling; TIM-4 blockade addresses T-cell sequestration, whereas MerTK inhibition alters efferocytosis. None of these macrophage-directed approaches has been validated in CRCPM patients, and VSIG4 expression alone does not specify population function. Intraperitoneal messenger RNA (mRNA) nanoparticles delivered chimeric antigen receptor macrophages and enhanced control of CT26 tumors when combined with PD-1/PD-L1 blockade (60). This strategy represents local myeloid engineering rather than T-cell ACT or correction of an endogenous metabolic state.

Whether a target is desirable also depends on when and where the intervention can reach it. Free cavity cells, ascites macrophages and macrophages embedded in fibrotic omental nodules experience different drug exposures; an intervention that helps during seeding may fail after lesions are established. Serial tissue and ascites sampling during oxaliplatin-based pressurized intraperitoneal aerosol chemotherapy showed reduced hypoxia-associated signals and stronger lymphocyte and tertiary lymphoid structure signatures (61). Because ICB was not administered and macrophage mediation was not demonstrated, the study motivates combination trials rather than proving sensitization. Serial sampling across peritoneal compartments during ICB is a translational priority. Ascites, omentum and solid lesions should be collected before treatment, during response and at progression, recording MMR/MSI status, CMS, ascites volume, disease burden and prior therapy. Pairing single-cell profiles with spatial imaging, metabolite measurements and ex vivo perturbation would connect state abundance and target engagement to metabolic change, CD8^+^ activity and clinical outcome. Longitudinal sampling can help distinguish treatment-induced change from selective enrichment, but establishing mechanism still requires lineage-informed and functional analyses. Until then, macrophage programs are candidate modifiers of responsiveness, not validated biomarkers of acquired resistance.

## 7 Conclusion

Macrophage states should be interpreted alongside, not instead of, MMR/MSI-based stratification. They may add a compartment- and stage-specific regulatory layer to immunotherapy responsiveness in CRCPM. Lipid-related programs can support or restrain T-cell immunity, whereas surface markers such as VSIG4 are insufficient on their own to define macrophage function. The SPP1 literature combines a resistance-associated human state with causal ICB/ACT findings from preclinical models, but cross-sectional patient data and unresolved SPP1 routes leave the clinical connection incomplete. Translational studies should test function-selective interventions in defined compartments and treatment windows while determining whether early protective macrophage activity can be preserved.

## Supplementary material

Supplementary Figure 1 Compartment-associated macrophage states and divergent immune effects in CRCPM. Peritoneal fluid or ascites, the omental adipose niche and solid lesions can coexist; the schematic does not imply a fixed lineage or temporal progression. Human studies identify C1Q+ and SPP1+ states with partly shared markers, including VSIG4 (22, 33). Lipid-related pathways involving ABCA9-dependent cholesterol handling, tumor-derived LPA signaling and TLR4–CXCL10 activation support T-cell-mediated tumor control in premetastatic or early-seeding models without testing ICB or ACT (39–41). In established murine disease, macrophage-intrinsic SPP1-associated PPARγ programming and extracellular SPP1–CD44 signaling constrain CD8+ T-cell function. SPP1 targeting improves ICB and ACT efficacy, but the contribution of each route to treatment rescue remains unresolved (33). TIM-4-mediated T-cell retention and MerTK-mediated efferocytosis represent parallel non-metabolic constraints (51, 52); the MerTK vaccine study tested a separate treatment endpoint (53). H denotes human observational association; M denotes a preclinical mechanism without an ICB/ACT endpoint; T denotes a preclinical intervention that alters ICB/ACT efficacy. These codes describe the evidence supporting the indicated regions and links, not fixed categories assigned to molecules. LPA, lysophosphatidic acid; ICB, immune checkpoint blockade; ACT, adoptive cell therapy.
